# Participatory development of an evaluation and data model for teleconsultations in long-term care: study protocol based on the MRC framework

**DOI:** 10.1136/bmjopen-2025-107644

**Published:** 2026-01-23

**Authors:** Sofie Woessner, Laura Hahn, Christina Kaltenbach, Stefanie Joos, Cornelia Mahler, Cornelia Henschke, Roland Koch

**Affiliations:** 1 Institute of General Practice and Interprofessional Care, University Hospital Tuebingen, Tuebingen, Germany; 2 Department of Nursing Science, Institute of Health Science, University Hospital Tuebingen, Tuebingen, Germany; 3 Department of Primary Care, University of Marburg, Marburg, Germany

**Keywords:** Implementation Science, Digital Technology, Telemedicine, HEALTH ECONOMICS, Nursing Homes, Nursing research

## Abstract

**Abstract:**

**Introduction:**

Demographic change is resulting in a growing number of individuals requiring nursing care, while the availability of professional caregivers is simultaneously declining. This imbalance places increasing pressure on care provision, particularly in home settings and rural areas. Primary care services are also under pressure. Digital solutions such as teleconsultations are considered promising strategies to support intersectoral collaboration in long-term care and to mitigate existing gaps in service provision.

**Methods and analysis:**

Seven teleconsultation projects in long-term care are being conducted using a mixed-methods design aligned with the phases of the Medical Research Council framework: with a focus on the feasibility phase. Data on structural conditions, usage patterns and user acceptance will be collected through standardised instruments (including Unified Theory of Acceptance and Use of Technology and Fit between Individuals, Task and Technology). In addition, focus groups and interviews will be carried out. The quantitative analysis will include descriptive and inferential statistical methods and will be complemented by a cost–consequence analysis. The qualitative data will be analysed using structuring content analysis. The aim is to provide a context-informed and theory-informed assessment of the implementation and potential impact of telemedical consultations.

**Ethics and dissemination:**

The study has been approved by the Ethics Committee in Tübingen. Participation is voluntary and based on written informed consent. Data protection is ensured in accordance with the GDPR (General Data Protection Regulation); all data are pseudonymised and processed separately. Results will be communicated in a target group-appropriate manner and published in scientific journals. Practice-oriented recommendations will be developed to support the further advancement of telemedical care in nursing.

**STRENGTHS AND LIMITATIONS OF THIS STUDY**

- This study will apply a theory-based, mixed-methods approach grounded in the Medical Research Council framework for complex interventions, incorporating participatory instrument development and context-sensitive data collection.
- Although this study does not aim to evaluate effectiveness, it will systematically prepare the ground for a future outcome-focused trial by developing core data structures and measurement tools.
- The German translations of Unified Theory of Acceptance and Use of Technology (UTAUT) and UTAUT2 instruments used for measuring technology acceptance are only partially validated, which may limit the comparability of findings.
- Due to data protection constraints, patient-related data will be collected in an aggregated form, which may limit the depth and granularity of individual-level analyses.

## Introduction

### Background and problem definition

 Current projections on demographic change indicate that the number of individuals with complex chronic conditions who require nursing care will continue to rise, while the number of professional nursing staff will decline at the same time.^1^ In 2023, a total of 5.7 million people in need of care were supported by 1.25 million caregivers, with the majority of care recipients receiving care in their own homes.^2^

International comparisons suggest that the German healthcare system is, to date, only partially able to respond adequately to the growing demand for nursing care.^3^ This becomes evident in various indicators. For instance, 39% of affected households report substantial difficulties in financing nursing services, which is above the European average.^4^ In addition, only about one quarter of elderly people with severe functional limitations receive formal nursing care, with rural regions in particular being undersupplied due to structural deficiencies.^5^ Public perceptions point to deficits in fairness and accessibility. In a 2019 EU-wide population survey, Germany received a rating of 7.2 out of 10 for perceived fairness of access to health and long-term care services, which is below the EU27 average and considerably lower than in countries such as Sweden or Denmark.^6^ The increasing demand for nursing care also places significant strain on long-term care systems. According to a report by the OECD (Organisation for Economic Co-operation and Development) and the European Commission, average expenditure on long-term care in OECD countries was 1.6% of GDP (Gross Domestic Product) in 2010 and is expected to double by 2050. Furthermore, public spending on long-term nursing care across 25 OECD countries increased by 9% per year, compared with 4% for general healthcare expenditure.^3^ At the same time, primary care is similarly affected by demographic dynamics, resulting in a high workload for general practitioners (GPs). In 2020, more than one-third of GPs in Germany were aged over 60, contributing to a growing need for health professionals to ensure nationwide coverage, particularly in structurally disadvantaged rural regions.^7^ The resulting situation in nursing care is highly challenging in light of increasing multimorbidity and underscores the need for coordinated support across healthcare sectors.^8^

### Teleconsultations as a potential solution

Digitalisation approaches to improving the care situation, such as teleconsultations, have increasingly been shown in studies to have a positive impact at the patient level.^9^ These approaches are supported by the telematics infrastructure,^10^ which aims to create the conditions for cross-sectoral and cross-system information exchange. The Digital Healthcare Act (DVG) provides the basis for promoting the digitalisation of the healthcare system in Germany by supporting the use of digital health applications, telemedicine and the expansion of the telematics infrastructure.^11^ The German Medical Association describes telemedicine as involving the direct or indirect participation of physicians in diagnostics, therapy and rehabilitation.^12^ In some cases, a video consultation is also offered, referred to here as a teleconsultation.^13^ As early as the end of 2016, the National Association of Statutory Health Insurance Physicians (KBV) and the National Association of Statutory Health Insurance Funds (GKV-Spitzenverband) formulated an agreement intended to ensure technical security and data protection for both medical professionals and patients.^14^ International examples show that successful digitalisation requires clear governance, standardisation and targeted funding, regardless of a country’s resource level.^15^ Countries such as the USA, the UK and France are ahead of Germany in terms of interoperability and the implementation of electronic patient records.^16 17^ The USA in particular combines technical standards with fast reimbursement models, serving as a model for app approvals under the DVG.^18^ In Germany, there is growing commitment to practical implementation, for example through model teleconsultation projects in long-term care.^19^ This study protocol outlines the planned evaluation of telemedical approaches implemented in seven projects introducing teleconsultations, funded by the Ministry for Social Affairs, Health and Integration Baden-Württemberg. The technical approaches applied across the seven projects can be categorised into three basic typologies based on their technological design and implementation.

#### Comprehensive solutions for real-time teleconsultations

Preconfigured systems with integrated hardware and software for synchronous video consultations, including certified software as well as medical equipment such as digital stethoscopes, ECG or ultrasound devices.^11^

#### Platform-based solutions without integrated medical technology

Web-based or cloud-based platforms for video consultations without integrated medical peripherals. Users provide their own camera, microphone and related medical equipment individually.^20^

#### Asynchronous, message-based communication

Time-delayed communication via structured text, image or file messages that can be flexibly integrated into daily workflows, without fixed appointments or video connections.^11^

### Theoretical and conceptual framework

This study protocol follows the Medical Research Council (MRC) framework for complex interventions. Phase 1 (Development) involves the theoretical development of the complex intervention, taking into account the context and relevant influencing factors. The results of this phase will be presented in the preliminary results. The study is primarily situated in phase 2 (Feasibility) of the MRC framework for complex interventions.^21^ We aim to assess the practical implementation, acceptance and contextual fit of teleconsultations in long-term care. While the teleconsultation models themselves are developed and implemented independently by the participating projects, the interest-holder mapping, evaluation logic and data collection instruments will be developed through a participatory, theory-informed process. This includes codesign workshops, a contextual interest-holder analysis and iterative pre-testing of the measurement tools. The term ‘interest-holder’ is chosen as a more inclusive and accurate alternative to ‘stakeholder’, emphasising direct interest and avoiding colonial connotations.^22^

Within the feasibility phase, a formative evaluation will be conducted to explore implementation-related factors such as usability, organisational effort and interest-holder-perceived benefits. Although no summative effectiveness evaluation will be carried out at this stage (phase 3), selected outcome-related data will be collected to inform future trial design.

In addition, the study reflects key elements of phase 4 (Implementation), as we generate decision-relevant evidence for potential transfer into standard care. This includes empirical insights into system-level requirements such as staffing capacity, training needs; variability in organisational settings and provider types; as well as interest-holder perspectives on resource allocation and long-term acceptability. These aspects are essential for understanding the conditions under which teleconsultations can be sustainably integrated into routine care structures.

The theoretical foundation of this study is based on a triangulated conceptual framework that integrated three established models: the FITT model (‘Fit between Individuals, Task and Technology’),^23^ the DeLone & McLean model for evaluating information system success^24^ and the UTAUT2 model (‘Unified Theory of Acceptance and Use of Technology’).^25^,^27^ The FITT model provides a sociotechnical baseline perspective by emphasising the fit between individuals (eg, nurses, physicians, patients), the tasks to be supported (eg, medical and nursing documentation, teleconsultation), and the technologies employed.^23^ This structural perspective is complemented by the DeLone and McLean model, which conceptualises implementation as a process of success driven by interrelated causal mechanisms.^24^ Finally, the UTAUT2 adds a behavioural dimension by explicitly integrating key determinants of technology acceptance and usage in healthcare settings.^27^

### Study aim

The present study aims to systematically capture, analyse and evaluate the early implementation and contextual integration of telemedical consultations in long-term care, considering the complex contextual conditions of this setting. Following the MRC framework for complex interventions, the study is mainly positioned at the interface of Feasibility and Evaluation, while delivering key insights to inform Implementation phase. The current focus lies in applying this framework to accompany the introduction of teleconsultations in practice and to assess their implications.

Central research questions:

Phase 1: What evidence-based components can be identified through a participatory interest holder analysis to inform the design of a contextually tailored evaluation for teleconsultations in long-term care settings?

Phase 2: To what extent can context-specific insights gained during the feasibility phase inform the practical implementation of teleconsultations in long-term care?

Phase 3: How do teleconsultations interact with the organisational and interprofessional context of ward rounds in long-term care settings, and what are their costs and consequences for care delivery?

#### Study objectives

- Participatory development of data collection instruments and data flow processes for the evaluation of teleconsultations tailored to the long-term care context.
- To identify organisational, technical and contextual factors influencing the implementation of teleconsultations in long-term care.
- Assessment of acceptance and perceived usefulness of teleconsultations among nursing professionals, affiliated physicians and other key interest-holders.
- Analysis of interprofessional collaboration, with a focus on how teleconsultations shape interprofessional ward round practices.
- Systematic assessment of direct and indirect costs associated with implementing teleconsultations.
- Cost–consequence analysis (CCA) of teleconsultations within interprofessional ward rounds in long-term care.

## Methods and analysis

### Study context, complexity and setting

**Figure 1:**
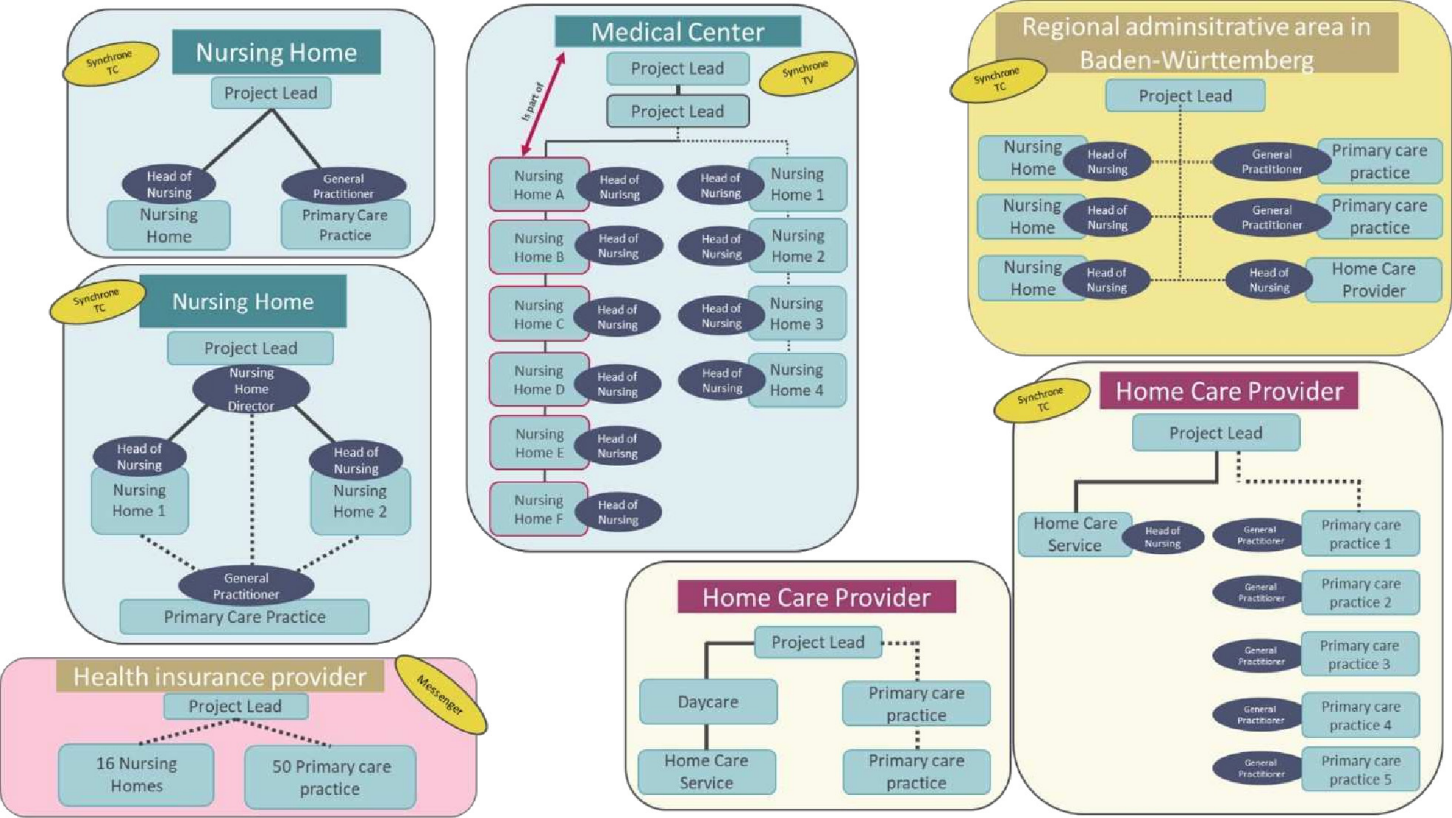
Overview of project structures: Colour coding indicates the care setting: blue, partially inpatient or inpatient; light yellow, outpatient; care, mixed setting; pink, health insurance provider. Light blue rectangles represent project leads. Dark blue ovals indicate key contacts relevant to implementation and data collection. Solid lines indicate direct organisational affiliation. Dashed lines indicate loose connections or connections limited to the project context. TC, Teleconsultion; TV, Televisit (Own illustration).

As part of a funding initiative by the Ministry of Social Affairs, Health and Integration of Baden-Württemberg, seven distinct projects are implementing teleconsultations in inpatient and outpatient long-term care settings across the region. Given the diverse technical, organisational and institutional starting conditions across these projects, a structured visual representation was developed to support the interpretation of project contexts (see figure 1). The figure serves as an analytical aid by illustrating how each project is embedded within existing care structures and by providing an overview of key interest-holders and potential data flows. It also reflects the heterogeneity of the telemedical solutions used, ranging from fully equipped systems for synchronous teleconsultation to modular platform solutions and asynchronous messenger-based formats.

This differentiation is important to understand structural and functional variation across the projects, as it may directly influence implementation strategies, interest-holder engagement and data management. The heterogeneous setting involves multiple interrelated factors, including varying degrees of interest-holder involvement (eg, care staff, physicians and project managers with different roles and levels of acceptance), challenges in technological integration and usability, differences in organisational structures (such as ownership models, facility sizes and infrastructure), and sociocultural aspects such as attitudes towards digital innovation and interprofessional collaboration.

In response to this complexity, the study adopts a context-sensitive research strategy. It uses data collection instruments that are usable in each unique project setting, while ensuring generalisability of findings. The schematic representation thus serves as a structural point of orientation within the diverse project contexts and provides a conceptual framework for the subsequent methodological approach. It highlights key contextual conditions, potential interfaces and data flow that are relevant for the analysis and classification of teleconsultation processes.

### Study design

Methodologically, a triangulated mixed-methods approach will be employed, integrating qualitative and quantitative methods to systematically capture processes, structures and perceived effects.

**Figure 2:**
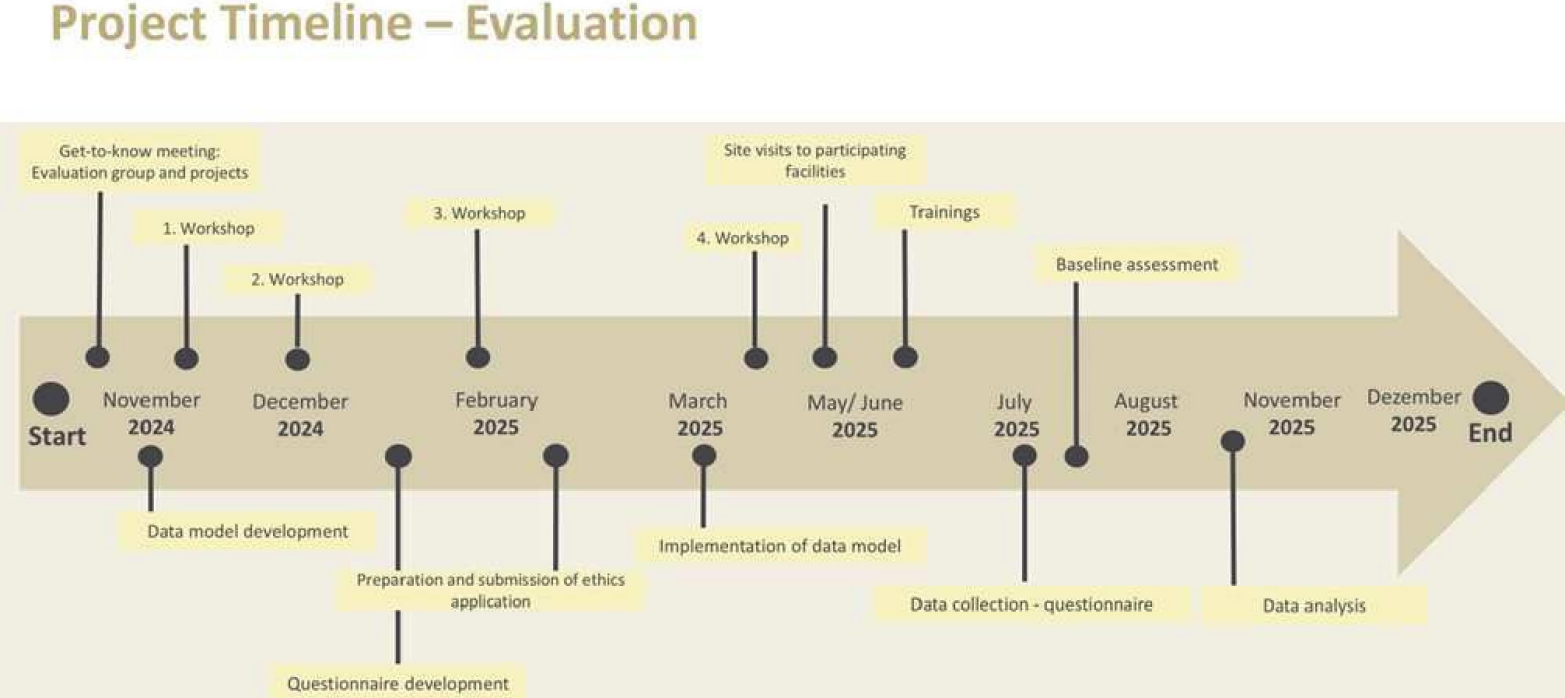
Project timeline.

The evaluation process began in autumn 2024 with a participatory interest-holder analysis (see figure 2). In several facilitated workshops with representatives from all seven projects, technical, organisational and care-related preconditions were systematically recorded. In addition, key dimensions were jointly identified, prioritised and operationalised, forming the basis for a shared and transferable data model. The participatory interest Stakeholder analysis involved the project leads from all seven projects. Project leads also invited selected staff members from their respective project contexts, including care facility managers, project managers, registered nurses, GPs, administrative staff and medical assistants (MFAs), to contribute to the workshops.

In June 2025, a baseline assessment will be conducted to capture the initial status of the relevant variables identified. Based on this, four follow-up measurements will take place from July to September 2025, at regular intervals of 4 weeks, to systematically document developments and changes over time. Between August and September 2025, focus groups will be conducted to gain insights into participants’ experiences and perspectives. The final analysis of the collected quantitative and qualitative data will take place between October and December 2025.

### Description of the intervention

Within the scope of the funded programme, teleconsultation will be independently implemented within the participating projects. To evaluate the implementation of teleconsultation in inpatient and outpatient long-term care facilities, it will be necessary to develop a common and transferable data-collection logic. Due to differing models of care, technical infrastructures and organisational conditions within the seven projects, a structured, participatory development process was initiated, which constitutes a methodological component of the study design. The aim is to define valid, comparable and practicable data flows that enable a meaningful evaluation. Representatives of the participating projects are referred to as interest-holders,^22^ as defined above.

At the outset, technical, organisational and structural care characteristics of the participating projects will be recorded to identify differences and commonalities. This initial problem assessment prepares the subsequent coordination process and establishes a shared understanding of the diversity of baseline conditions. Based on this, four digitally facilitated interest-holder meetings were held between November 2024 and March 2025, in which overarching goals for the evaluation were discussed and jointly agreed on.

This interest-holder analysis was based on a contextual interest-holder analysis according to Varvasovszky and Brugha.^28^ In view of the structural and organisational heterogeneity of the participating projects, this methodological approach was chosen to systematically capture both the potential influence and the degree of involvement of the different target interest-holder groups. The interest-holder groups derived from this analysis will form the basis for the subsequent participatory development process of the evaluation.

The projects can contribute their expertise as interest-holders in context-specific care models, particularly regarding technical feasibility, organisational prerequisites and the relevance of potential data collection content.

Building on this shared understanding, a data model will be developed that, among other elements, includes usage frequency, structural characteristics and economic parameters. Standardised data collection instruments, such as questionnaires and documentation tools, will be derived from this model in an iterative process. These instruments will then be refined through multiple feedback loops with the interest-holders, with particular attention on comprehensibility, relevance and feasibility.

In addition to this development process, site visits will be conducted at selected facilities to observe actual teleconsultation procedures. The practical insights gained from these observations inform the linguistic and functional design of the instruments, improving alignment between the data collection logic and real-world care settings. In a final pre-test, the completed instruments, comprising four questionnaires and supplementary completion aids, will be made available for review through the interest-holders and subsequently revised based on the feedback received.

### Target groups

The target groups of the study will include key professional groups who will be involved in the planning, implementation and use of teleconsultation in long-term care. These will be:

- Nursing staff in inpatient and outpatient care facilities.
- GPs, regardless of their organisational setting (solo practice, medical care centres, or employed within institutions).
- Non-physician healthcare professionals working in general practice teams, particularly MFAs, non-physician practice assistants, care assistants in general practice (VERAHs) and physician assistants (PAs), who will contribute to shaping telemedical rounds from an organisational, technical and, in some cases, clinical perspective?
- Management and coordination staff from care facilities or outpatient services.
- Residents with the capacity to give consent who will be directly involved in the teleconsultation.

A priori sample size calculation will not be conducted, as the telemedical round will represent a complex, context-dependent and practice-oriented care model.^27^ The projects will differ significantly in terms of structures, target groups and implementation strategies. Given this heterogeneity and the exploratory nature of the study, data collection will be based on the actual number of cases (teleconsultations) available within the participating projects.

Recruitment will be carried out within the seven projects, following a theoretically grounded, pragmatic sampling approach.^29^ Inclusion and exclusion criteria will be based on actual involvement in the teleconsultation process, the functional role within the facility or practice, and the capacity to provide informed consent.

### Data collection

To enable a comprehensive analysis of the telemedical rounds, different types of data and methodological approaches will be combined. Data collection will follow a triangulated, cross-project design that allows for comparability and is structured along two data collection strands (table 1). On the one hand, structural context data will be collected once at the beginning of the data collection phase. On the other hand, aggregated usage data and standardised questionnaires will be collected at three measurement points, each spaced 2 months apart.

**Table 1:** Overview of data collection instruments and measurement time points

Data type/collection method (instrument)	Baseline (T0)	Follow-up 1 (T1)	Follow-up 2 (T2)
Structural context data (standardised questionnaire)	X		
Aggregated usage data (standardised data collection form)	X	X	X
Subjective experiences (standardised questionnaire)	X	X	X
Success factors and challenges/FITT-based interview guide (qual.)			X

FITT, Fit between Individuals, Task and Technology.

#### The quantitative data collection will comprise

- Structural context data of the participating provider organisations, including the number of sites, staffing ratios, ownership type, geographic location and infrastructural connectivity. These data will serve to contextualise the project sites and to describe the structural framework conditions.
- Aggregated usage data relating to the number of teleconsultations performed, the type and frequency of technical disruptions, as well as the documented contents and outcomes. In addition, data on costs and implementation efforts will be collected, including time requirements, human-resource requirements, technical support needs and procurement channels. Data collection will be standardised via the project leads to enable a comparative analysis of practical implementation across participating sites.
- Standardised questionnaires on the subjective experiences and acceptance of the participating interest-holders. Based on the UTAUT2 model, individual assessments, frequency of use and perceived usefulness will be recorded.

#### Data collection instruments used

- Standardised data collection form for aggregated usage data (completed by project leads); three data collection points within a 3-month period.
- Questionnaire on individual acceptance and usage for nursing staff, GPs and other healthcare professionals; three data collection points within a 3-month period.
- FITT-based interview guide used for conducting focus groups; single data collection point.

#### The qualitative data collection comprises

- Focus groups with interprofessional teams and semistructured interviews will be conducted to gain deeper insights into experiential contexts, success factors and challenges in the application of telemedical solutions.^30^

### Outcomes

The evaluation aims to achieve a comprehensive understanding of the use, acceptance and effects of teleconsultation in long-term care. Outcome measurement is theory-driven (FITT and UTAUT2) and considers economic, process-related and subjective impact levels.

#### Costs and economic consequences

Both direct and indirect costs will be recorded. In addition, perceived benefits such as time savings or improved coordination will be documented. The presentation follows a CCA.

#### Structures and processes

Structural characteristics of the participating facilities (eg, ownership, staffing ratios, connectivity) will be collected, along with process-related changes in day-to-day care . These include, among others, communication frequency, coordination effort and the temporal sequence of rounds.

#### Usage and feasibility

The practical use of telemedical rounds will be described using aggregated usage data (eg, number and duration of sessions, technical disruptions, documented content). In addition, the perceived fit between technical requirements, task profiles and individual preconditions is analysed, based on the FITT model.

#### User acceptance and perceived benefit

Individual acceptance of teleconsultations will be examined using the UTAUT2 model capturing constructs such as Performance Expectancy, Effort Expectancy, Social Influence and Facilitating Conditions. Furthermore, usage experiences, satisfaction and the subjectively perceived benefit will be considered according to the DeLone & McLean IS Success Model.

### Data analysis

Data analysis follows a mixed-methods approach based on the collected quantitative and qualitative data sources and includes a separate economic assessment in the form of a CCA.

#### Quantitative data analysis

##### Survey data collection

Statistical analysis is performed using R (V.4.4.1), employing the packages gtsummary (V.2.2.0) and stats (V.4.4.1). Quantitative data from standardised questionnaires on individual use and acceptance (nursing staff, physicians), as well as from standardised forms on aggregated usage data, are first analysed descriptively (means, measures of dispersion, frequencies). In addition, regression analyses are used to examine potential associations between individual user characteristics and the core constructs of the UTAUT2 model. The goal is to identify relevant factors influencing the acceptance of telemedical consultations. The questionnaires used (excluding the UTAUT2 instrument) are provided as online supplemental material. The UTAUT2 questionnaire will be made available separately (onlinesupplemental materials 1^4^).

##### Cost–consequence analysis

As part of the CCA, relevant cost categories will be identified. The quantities of resource consumption will be recorded and corresponding unit costs or prices are assigned.^31 32^ Based on a cost–consequence approach, data on implementation and operational costs, as well as associated consequences, will be collected and recorded in order to enable a transparent comparative assessment. The analysis distinguishes between the following types of costs: direct costs, such as personnel and technology costs; indirect costs, such as training expenses, documentation effort and coordination activities; and intangible costs, such as changes in team climate. In addition, costs will be categorised by phase of occurrence (implementation costs vs operational costs), reflecting when and where the effort arises.

Consequences will include both health-related outcomes (eg, avoided hospital admissions, based on a self-developed questionnaire) and non-health-related outcomes (eg, technology acceptance, based on UTAUT2). In addition, perceived benefits from the perspective of interest-holders will be collected (eg, facilitation of care delivery, time savings, improved interprofessional communication). The aim is to make different cost–consequence relationships visible without monetising the effects. For further statistical analysis, inferential methods will be applied to test significant differences. Significance testing will be conducted at a level of α=0.05. Depending on the time points of measurement, the scale level and the data distribution, either repeated measures analysis of variance or a linear mixed model will be applied.

The CCA will be conducted as part of the formative evaluation. While no control group is included and effects are not monetised, the CCA is designed to provide a structured overview of implementation-related costs and interest-holder-perceived consequences—such as time savings or improved care coordination. This supports a deeper understanding of contextual resource requirements and lays the foundation for future summative and economic evaluations.

### Qualitative data analysis

The qualitative data from focus groups and semi-structured interviews are analysed using structuring content analysis according to Kuckartz.^33^ Categories are developed deductively based on the FITT perspective (fit between individual, task and technology) and are extended inductively as needed. The aim is to systematically capture subjective experiences, facilitating factors, barriers and contextual influences. The analysis is supported by software (eg, MAXQDA or comparable tools). The interview guide used is available in translated form as online supplemental material 5.

### Patient and public involvement

Patients and members of the public were not involved in the design, conduct, or reporting of this study protocol, and no such involvement is planned. The evaluation and data model was developed using a participatory approach with professional interest-holders involved in the project.

## Preliminary results: learnings from the interest-holder analysis

As part of four interest-holder workshops, representatives from all seven projects were continuously involved, including nurse managers, GPs and MFAs (n=average 15–20). The workshops supported a structured discussion of goals, types of data and responsibilities, aligned with the three evaluation dimensions: structure, process and outcome, and at the same time facilitated exchange among project participants. A key result of this participatory phase was the project-specific adaptation of the questionnaire logic, designed to reflect the heterogeneity of the settings (see online supplemental appendix). Furthermore, the collaboration fostered a relationship of mutual trust between the projects and the evaluation team, which is considered beneficial for the subsequent data collection phase.

**Figure 3:**
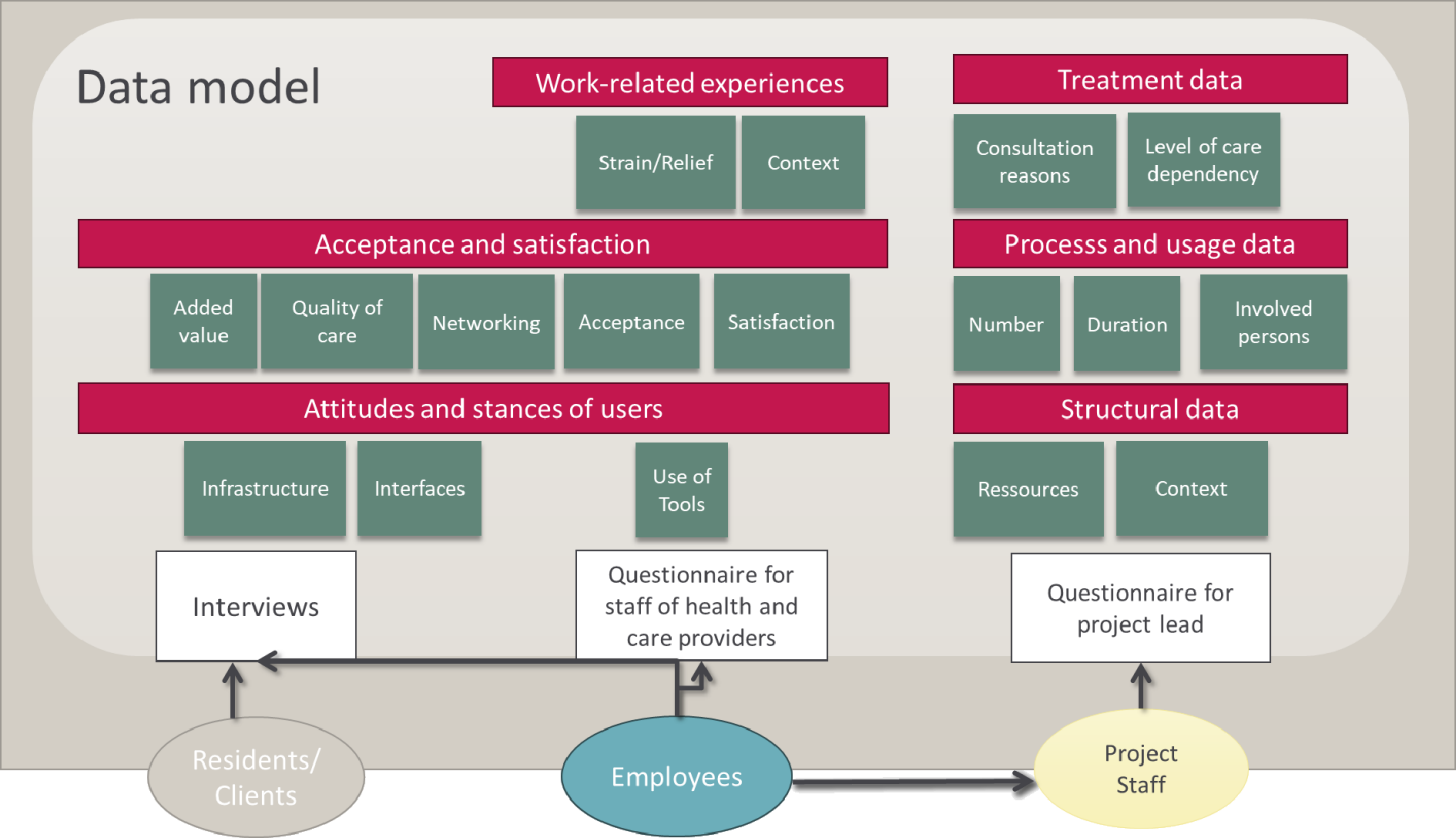
Developed data model.

Due to the heterogeneity of the participating pilot projects, a data model developed jointly and participatively with the project partners is used^34^ (see figure 3). This model enables a tailored assessment of the target groups regarding various cost and consequence categories and incorporates both quantitative and qualitative data. The results of the interest-holder workshops form the basis for the development of a shared data model and for the operationalisation of standardised evaluation parameters. In the second phase, a formative evaluation is conducted, systematically capturing and analysing objective outcome data as well as subjective experiences. The expected results of the study will be presented in a disaggregated format to provide a transparent and differentiated foundation for decision-making in the further development of teleconsultation services in long-term care.

## Discussion

### Potential impact

This development process provided the methodological foundation for a theory-based, context-sensitive and practice-oriented evaluation of highly heterogeneous projects under complex conditions. In line with this focus, the protocol does not aim to evaluate effectiveness. Rather, it details the participatory development of the data model and data collection instruments that will underpin the planned analyses in the next phase of the study. It ensures practical relevance, enhances data quality through shared understanding of the relevance and measurability of key aspects, and embeds the perspectives of the participating projects as a formative element of the study design.

The close involvement of project coordinators and other interest-holders is in line with the MRC framework’s recommendation to evaluate complex interventions through iterative, participatory processes.^21^

In this study, the early and deliberate involvement of interest-holders proved beneficial for the analysis process. At the same time, it also entailed a responsibility to maintain ongoing dialogue with project partners, to incorporate their feedback while maintaining the independence and neutrality of the evaluation. The participatory workshops played a key role in developing a context-sensitive and practice-oriented data model as well as target group-appropriate questionnaires. This co-creative approach not only increased the relevance and acceptance of the instrument but also supported implementation across heterogeneous care settings. A major advantage lies in the improved content validity and closer alignment of data collection with care practice. However, this is balanced by the increased organisational effort and the challenge of integrating diverse perspectives into a coherent evaluation design.^28^

A key insight was that interest-holders in long-term care can act not only as a target group or data source, but also as co-designers of the evaluation. They actively contributed to the structure of the data model (eg, by selecting practice-relevant indicators) and to the wording of the questionnaires (ie, ensuring clarity and feasibility in everyday care settings). This enhanced the transferability between data collection instruments to real-world care environments, but also increased the complexity of the data structure.

In comparison with existing literature on teleconsultations and interest-holder involvement, there is increasing recognition of multiphase, participatory research approaches. For example, Khanassov *et al*,^35^ in their telemedicine study in a primary care setting involving older patients, emphasise the importance of a structured, iterative process across multiple phases in order to address both technical and contextual challenges at an early stage. Similarly, Piau *et al*,^36^ in the DETECT study, a cluster-randomised design in residential care facilities, highlight the relevance of feasibility assessments and context-sensitive implementation strategies, particularly in dealing with neuropsychiatric symptoms among geriatric residents. Both studies underscore the need to conceptualise telemedical interventions not only in technical terms but also from organisational and interdisciplinary perspectives. This is a perspective that our participatory approach consistently embraces and adapts to the specific characteristics of long-term care.

### Ethics

The study was approved by the responsible ethics committee of the Eberhard Karls University of Tübingen (Project number 110/2025). Participation in all data collection activities (interviews, focus groups and online surveys) is voluntary and requires written informed consent. Depending on the mode of data collection, different GDPR-compliant data protection measures (General Data Protection Regulation) are applied, including participant-managed pseudonymisation codes for online surveys. Personal data are processed strictly separately from study data and are not linked to particularly sensitive information. Data processing is carried out exclusively on ISO 27001-certified systems. The online surveys are conducted using the SoSci Survey platform, which meets all required security and data protection standards. This is a non-interventional observational study that does not interfere with existing care or implementation processes. Potential burdens due to time requirements are mitigated through participatory design of the data collection instruments and flat-rate compensation for participants. Additionally, modifications to the data collection instruments resulting from interest-holders’ feedback are implemented and submitted to the ethics committee as an amendment.

### Dissemination

The study results will be prepared in a differentiated and target group-oriented manner. Feedback will be provided in a structured form to the participating care facilities, project sponsors and the Ministry of Social Affairs, Health and Integration of Baden-Württemberg. The findings will include both process-related results and information on the costs and effects of the various teleconsultation models. For academic dissemination, the publication of a study protocol and the presentation of the mixed-methods findings in peer-reviewed journals and at scientific conferences are planned. In addition, the development of practice-oriented recommendations forms a central part of the dissemination strategy. These recommendations are intended to provide care providers and policymakers with an evidence-based foundation for further developing teleconsultations in the long-term care sector.

### Advantages and weaknesses of this study

Compared with existing literature on teleconsultations and interest-holder engagement, this study places greater emphasis on interprofessional collaboration and participatory development processes, the research team consisting of academic qualified GP, nurse, health services researcher and health economist. Organisational, communicative and cultural factors are brought into focus, as human resources and digital infrastructure are often limited in healthcare settings. The strengths of this approach include comprehensive consideration of context, strong practical relevance and the iterative refinement of the study design in close collaboration with care practice. Limitations include the high coordination effort required, potential bias due to particularly active or dominant interest-holders and limited generalisability of findings to other care settings. Due to the project’s framework conditions, particularly data protection regulations and the limited project duration, it was not possible to collect or analyse individual-level personal data. Only aggregated data precompiled by participating care facilities or IT service providers will be collected. In a CCA, it is not necessary to select a single outcome or consequence, which makes it a suitable method for this study.^32 37^

## Supplementary material

10.1136/bmjopen-2025-107644 online supplemental file 1

10.1136/bmjopen-2025-107644 online supplemental file 2

10.1136/bmjopen-2025-107644 online supplemental file 3

10.1136/bmjopen-2025-107644 online supplemental file 4

10.1136/bmjopen-2025-107644 online supplemental file 5
