# Supplementary material for: Participatory development of an evaluation and data model for teleconsultations in long-term care: study protocol based on the MRC framework
Source: BMJ Open. 2026 Jan 23;16(1):e107644. doi: 10.1136/bmjopen-2025-107644 (PMC12853425; doi:10.1136/bmjopen-2025-107644)
Supplement: online supplemental file 2 [file bmjopen-16-1-s002.docx]

**Teleconsultation Survey - • Management and Coordination Staff**

**Welcome to our Management and Coordination Staff Survey!**

**Thank you for taking the time to participate in our survey.**

With this questionnaire, we want to learn more about teleconsultations in residential and home care facilities. This survey is part of a comprehensive evaluation of the use of teleconsultations in home care and long-term residential care.

**What is a teleconsultation?**

By teleconsultation, we mean a video conversation about a person under care. This could be a resident, patient, or client - depending on where you work.

Such conversations might involve nursing professionals, doctors, practice staff, family members, or other individuals.

**We want to find out:**

- How teleconsultations are currently used in practice
- What experiences you have had with them

**Who can participate?**

- Nursing professionals in nursing homes or home care
- Medical practice staff (e.g., Medical Assistants, VERAH, NäPA)
- Doctors and physicians
- Other individuals involved in teleconsultations

**Who are we?**

We are the team from the comprehensive evaluation of the Institute of General Practice and Interprofessional Care (IAIV) and the Institute of Health Sciences, Department of Nursing Science.

In the comprehensive evaluation, we scientifically examine the individual projects funded by the Ministry of Social Affairs.

If you have any questions, you can contact us at any time.

**Contact:** […]

**Important Information**

- Completion takes approximately 10 to 15 minutes
- Your information remains confidential and will be processed according to applicable data protection regulations. This means: Your answers will be securely stored, used only for evaluation, and not shared with third parties.

If you have any questions, you can contact us at any time

**Thank you for your participation!**

**Privacy Policy**

**Privacy Statement for Survey Participation in the Scientific Study:**

**Evaluation Project "Comprehensive Evaluation of Teleconsultations in Inpatient and Outpatient Long-term Care Facilities"**

**Information on Data Handling According to Article 13 EU General Data Protection Regulation (GDPR):**

**Survey Implementation:**

The surveys are conducted online using SoSci software. Completing the survey takes approximately 10-15 minutes. We conduct the survey in a pseudonymized manner: The surveys are marked with a personal code word (pseudonym). Only you can associate this pseudonym with your person. A guide for creating the code word is presented at the beginning of the survey. It is not possible for outsiders to trace the surveys back to your person. Only project staff from the comprehensive evaluation have access to the survey data.

Participation in the survey is fundamentally voluntary. You can withdraw your consent to participate at any time without giving reasons. Withdrawal has no disadvantages for you. All your information will be treated confidentially.

The processing, use, and archiving of pseudonymized data is done on survey forms and electronic data carriers. The pseudonymized data will normally be archived for 10 years after publication, unless the purpose of the study, e.g., for inclusion in a database and for long-term studies, requires a longer storage period.

The research results from the study will be published in anonymized form in professional journals or scientific databases. Your identity will not be revealed when publishing the research results.

You can request information about your stored data at any time and request a free copy, and you have the right to have incorrect data corrected. For this, you must personally contact the researchers and provide your pseudonym, because without the pseudonym it is not clear to the processing staff who you are. You can also request at any time that your data be deleted or anonymized so that a connection to your person can no longer be established. These rights are limited under § 13 of the State Data Protection Act insofar as these rights would likely make the realization of the respective research purposes impossible or seriously impair them, and the limitation is necessary for the fulfillment of the respective research purposes. The right to information also does not exist if the data is necessary for scientific research purposes and providing information would require disproportionate effort.

The data controller according to Art. 4 para. 7 GDPR is the University Hospital Tübingen, a legal entity under public law of the University of Tübingen, Geissweg 3, 72076 Tübingen, Phone: 07071 29-0, service@med.uni-tuebingen.de. The person responsible for data processing in this study is the study director Prof. Dr. Joos. If you have questions about the use or processing of your data, please contact Sofie Wössner (sofie.woessner@med.uni-tuebingen.de).

If you have concerns or complaints regarding data protection or want to exercise your rights according to Art. 15ff GDPR, you can contact: University Hospital Tübingen, Data Protection Officer, Geissweg 3, 72076 Tübingen, Phone: 07071 29-87667, Email: Datenschutz@med.uni-tuebingen.de. You also have the right to complain to the competent supervisory authority for data protection (State Commissioner for Data Protection and Freedom of Information in Baden-Württemberg, P.O. Box 10 29 32, 70025 Stuttgart, Phone: 0711 / 61 55 41 - 716, Email: Poststelle@lfdi.bwl.de).

The legal basis for processing is Art. 6 para. 1 lit. a General Data Protection Regulation (GDPR). Your express consent by signing the data protection consent form is required for the collection, storage, use, and disclosure of your data.

**Consent Statement for Handling Data Collected in the Study**

I agree that:

- Data about me will be collected, pseudonymized, archived, and possibly pseudonymized shared on electronic data carriers as part of this study
- The pseudonymized data may be used for publication purposes (including qualification papers such as dissertations). I was assured that all personal data that would allow conclusions about my person and my employer will be deleted or anonymized
- The pseudonymized data may also be used for teaching purposes and workshops in the Institute of General Practice and Interprofessional Care or in the Institute of Health Sciences, Department of Nursing Science, limited to small excerpts. Here too, I was assured that all personal data that would allow conclusions about my person and my employer will be deleted or anonymized
- The pseudonymized data may be used for further research projects of the Institute of General Medicine and Interprofessional Care or the Institute of Health Sciences, Department of Nursing Science

I have been informed that:

- I can request information about my stored data and correction of incorrect data at any time. For this, I must provide my pseudonym because otherwise a connection to my person is not possible
- I can request at any time, for example when withdrawing from study participation, that my data collected up to that point be deleted or immediately anonymized (unless there are restrictions according to § 13 of the State Data Protection Adaptation Act or § 27 of the Federal Data Protection Adaptation Act, see above)

I declare that I have been adequately informed about the collection and processing of my data collected in this study and my rights, and I agree to the use of the data collected in this study in the form specified above.

I am aware that implementation of my data subject rights is only possible by providing the pseudonym. I have been informed that I must keep the pseudonym in a safe place.

**Contact Information:**
[…]

**Consent Options:**

☐ No, I do not want to participate in this study.

☐ Yes, I agree to the use of data collected in this study in the described form. I know that I can request at any time, for example when withdrawing from study participation, that my data collected up to that point be deleted or immediately anonymized.

**Additional Contact Information:**

**Data Protection Officer of University Hospital Tübingen**
Calwerstraße 7/4, 72076 Tübingen
Phone: 07071 29-87667
Email: dsb@med.uni-tuebingen.de

**State Commissioner for Data Protection and Freedom of Information in Baden-Württemberg**
Postal Address: P.O. Box 10 29 32, 70025 Stuttgart
Phone: 0711/615541-0, FAX: 0711/615541-15
Email: poststelle@lfdi.bwl.de

**Pseudonym Creation**

**Why is your pseudonym important?**

Your pseudonym serves to ensure your anonymity in this survey. It will be assigned exclusively to you and will not be stored anywhere else or linked to personal data.

This ensures that your answers cannot be traced back to your identity.

Please keep your pseudonym carefully, as we will repeatedly ask you to provide the pseudonym during your survey.

You can find your pseudonym and its composition in the document you received with the privacy statement for the survey.

Please keep your pseudonym carefully. You will also need it for the next survey periods.

**Create Your Pseudonym:**

**The last two letters of your mother's last name:**[____]

**The number of letters in your mother's (first) first name (e.g., 08):**[____]

**The last two letters of your father's (first) first name:**[____]

**Your own birth date (only the day, e.g., 05):**[____]

**This is your pseudonym:**[________________]

Please keep your pseudonym carefully. You will need it on the next page and for the next survey periods.

**Pseudonym**

**1. Please enter your pseudonym here:**[________________]

**Personal Information**

**2. Please select the name of your project from the list!**

- ☐ AOK
- ☐ Bodenseekreis
- ☐ Diak Altenhilfe
- ☐ Diak Schwarzwald-Baar
- ☐ Kreiskliniken Reutlingen
- ☐ Sozialstation Sinsheim
- ☐ Wohlfahrtswerk

**Teleconsultation Usage Data**

**Questions on the Use of Teleconsultations**

In the following, we would like to ask you to answer questions about the **usage data of teleconsultations in the last four weeks**.

By the term **teleconsultations**, we understand any type of contact between those involved in the care process around the persons to be cared for, in which a **digital service** (e.g., synchronous video conference) is used.

**3. How many teleconsultation contacts were there within your project in the last four weeks?**

*Please provide a number. If you cannot provide information, note this in the text field.*

**Number:** _______________

**4. Please indicate how many teleconsultations were conducted in the last four weeks in the following categories.**

*Multiple selections possible. If no TV took place in the respective category, please enter zero.*

☐ **Emergency appointment:** ___________________________________________

☐ **Regular visit appointment** *(recurring doctor visit, usually routine)*: _________________________________________________________________

☐ **Scheduled appointment** *(specifically arranged appointment for a specific concern)*: _______________

☐ **Planned and did not take place** *(e.g., technical reasons, doctor unavailable, nursing staff unavailable, patient not available, short-term changes in daily routine, etc.). Please enter the reason*: ______________________________________________

☐ **No information possible/cannot be collected** *(e.g., no data basis available)*: __________________________________________________________________

**5. What were the most common reasons for a teleconsultation in the last four weeks?**

*You can select multiple answers. The symptoms mentioned here are to be understood as examples for the respective category. If you are unsure, choose the answer that fits best. Please also indicate the respective reason and how many people received a teleconsultation for this reason in the last four weeks.*

☐ **Mobility:** Person has difficulty walking, fall and/or fall consequences
☐ **Cognitive and communication abilities:** Person is disoriented. For example, doesn't recognize nursing staff or family members. Person can no longer express what they need
☐ **Behavioral patterns and psychological issues:** Person is aggressive, anxious, depressed, or confused. Nighttime restlessness or constant wandering, self-harming or endangering behavior, refusal of personal care
☐ **Self-care:** Person no longer eats or drinks adequately or too much. Can no longer manage personal hygiene or toilet visits independently. Problems with incontinence or hygiene
☐ **Managing illness or therapy-related requirements:** Acute or chronic wounds, acute or chronic illnesses, stoma or catheter care. Medication, vital signs, medical appointments (e.g., dialysis)
☐ **Palliative care:** End-of-life care, symptom management, consultation on further treatment
☐ **Other:** In addition to the above categories or none of the above categories applicable, but: [_____________]

**6. In how many cases was a hospital admission the result of a teleconsultation?**

*Please provide the exact number. If you don't know the exact number, please estimate it. If you cannot provide information, please note this as well.*

☐ **Exact number:** _______________
☐ **Estimated number:** _______________
☐ **No information possible**

**7. How long did all conducted teleconsultations last in total (sum in minutes) within the last four weeks?**

*Please provide the exact total duration. If you don't know the exact time, please estimate it. If you cannot provide information, please note this as well.*

☐ **Exact number:** _______________
☐ **Estimated number:** _______________
☐ **No information possible**

**8. How long did the shortest teleconsultation last (in minutes) that you conducted in the last four weeks?**

*Please provide the exact total duration. If you don't know the exact time, please estimate it. If you cannot provide information, please note this as well.*

☐ **Exact number:** _______________
☐ **Estimated number:** _______________
☐ **No information possible**

**How many people were usually cared for within one teleconsultation?** _______________

**9. How long did the longest teleconsultation last (in minutes) that you conducted in the last four weeks?**

*Please provide the exact duration. If you don't know the exact time, please estimate it. If you cannot provide information, please note this as well.*

☐ **Exact number:** _______________
☐ **Estimated number:** _______________
☐ **No information possible**

**How many people were usually cared for within one teleconsultation?** _______________

**10. Were teleconsultation tools (e.g., tele-EKG, tele-stethoscope, tele-ultrasound) used in the last four weeks?**

☐ **Yes**
☐ **No**
☐ **No information**

**11. How often were teleconsultation tools used in the last four weeks?**

*Please provide the exact number. If you don't know the exact number, please estimate it.*

☐ **Tele-EKG:** _______________
☐ **Tele-stethoscope:** _______________
☐ **Tele-ultrasound:** _______________
☐ **camera):** _______________

**12. Are your information about the numbers of teleconsultation tools exact (e.g., collected from the system) or based on an estimate?**

☐ **Exact information:** I added the frequency of teleconsultation tools used based on nursing documentation/tally list
☐ **Estimate:** I estimated the frequency of teleconsultation tools used

**13. In how many cases was medication changed as a result of a teleconsultation in the last four weeks?**

*Please provide the exact number. If you don't know the exact number, please estimate it. If you cannot provide information, please note this as well.*

☐ **Medications were added, how often:** _______________
☐ **Medications were discontinued, how often:** _______________
☐ **There was a dosage change, how often:** _______________
☐ **No change in the number of medications, how often:** _______________
☐ **No information possible, because:** _______________

**14. Are your information about the numbers of daily medication intake exact (e.g., collected from the system) or based on an estimate?**

☐ **Exact information:** I added the frequency of daily medication intake based on nursing documentation/tally list
☐ **Estimate:** I estimated the frequency of daily medication intake

**15. How frequently were the following care levels among the persons cared for with teleconsultations in the last four weeks?**

*Please provide the exact number for each care level. If you don't know the exact number, please estimate it.*

☐ **Care level 1:** _______________
☐ **Care level 2:** _______________
☐ **Care level 3:** _______________
☐ **Care level 4:** _______________
☐ **Care level 5:** _______________
☐ **No information possible, because:** _______________

**16. Are your information about the numbers of care levels exact (e.g., collected from the system) or based on an estimate?**

☐ **Exact information:** I added the frequency of care levels based on nursing documentation/tally list
☐ **Estimate:** I estimated the frequency of care levels

**17. How frequently were the following age groups among the persons cared for with teleconsultations in the last four weeks?**

*Please provide the exact number for each age group. If you don't know the exact number, please estimate it.*

☐ **18-29 years:** _______________
☐ **30-39 years:** _______________
☐ **40-49 years:** _______________
☐ **50-59 years:** _______________
☐ **60-69 years:** _______________
☐ **70-79 years:** _______________
☐ **80-89 years:** _______________
☐ **90-99 years:** _______________
☐ **100 years and older:** _______________
☐ **No information possible, because:** _______________

**18. Are your information about the numbers of age groups exact (e.g., collected from the system) or based on an estimate?**

☐ **Exact information:** I added the frequency of age groups based on nursing documentation/tally list
☐ **Estimate:** I estimated the frequency of age groups

**19. Please indicate how many persons to be cared for were assigned to which gender in the last four weeks within the framework of the teleconsultation?**

*Please provide the exact number for each gender category. If you don't know the exact number, please estimate it.*

☐ **Male:** **_______________**
☐ **Female:** **_______________**
☐ **None of the categories applicable:** _______________
☐ **No information possible, because:** _______________

**20. Are your information about the numbers of gender exact (e.g., collected from the system) or based on an estimate?**

☐ **Exact information:** I added the frequency of gender based on nursing documentation/tally list
☐ **Estimate:** I estimated the frequency of gender

**21. What technical problems occurred in the last four weeks?**

*Multiple selections possible.*

☐ **Image problems** (the image was blurry, jerky, or frozen)
☐ **Sound problems** (couldn't hear well, was too quiet, distorted, or choppy)
☐ **Complete connection failure** (the connection was completely lost, nothing worked anymore)
☐ **Unstable internet** (the connection was often poor or dropped out briefly multiple times)
☐ **Equipment failure** (a device didn't work)
☐ **Planned teleconsultation did not take place** (doctor couldn't make it, person in hospital)
☐ **Staff had problems with handling**
☐ **Patient/resident had problems with the application, device, or acceptance**
☐ **No problems** (everything worked well)
☐ **Other:** _______________
☐ **No information possible**

**Final Page: Thank You**

**Thank you for your participation!**

**Please keep your pseudonym carefully.**

**You will also need it for the next survey periods.**

If you have any questions or comments, please feel free to contact the following contact person:

[…]
