# Supplementary material for: Participatory development of an evaluation and data model for teleconsultations in long-term care: study protocol based on the MRC framework
Source: BMJ Open. 2026 Jan 23;16(1):e107644. doi: 10.1136/bmjopen-2025-107644 (PMC12853425; doi:10.1136/bmjopen-2025-107644)
Supplement: online supplemental file 3 [file bmjopen-16-1-s003.docx]

**Teleconsultation Survey - Medical Practice**

**Welcome to Our Survey – Medical Practice!**

**Thank you for taking the time to participate in our survey.**

With this questionnaire, we want to learn more about teleconsultations in residential and home care facilities. This survey is part of a comprehensive evaluation of the use of teleconsultations in home care and long-term residential care.

**What is a teleconsultation?**

By teleconsultation, we mean a video conversation about a person under care. This could be a resident, patient, or client - depending on where you work.

Such conversations might involve nursing professionals, doctors, practice staff, family members, or other individuals.

**We want to find out:**

- How teleconsultations are currently used in practice
- What experiences you have had with them

**Who can participate?**

- Nursing professionals in nursing homes or home care
- Medical practice staff (e.g., Medical Assistants, VERAH, NäPA)
- Doctors and physicians
- Other individuals involved in teleconsultations

**Who are we?**

We are the team from the comprehensive evaluation of the Institute of General Practice and Interprofessional Care (IAIV) and the Institute of Health Sciences, Department of Nursing Science.

In the comprehensive evaluation, we scientifically examine the individual projects funded by the Ministry of Social Affairs.

If you have any questions, you can contact us at any time.

**Contact:** […]

**Important Information**

- Completion takes approximately 10 to 15 minutes
- Your information remains confidential and will be processed according to applicable data protection regulations. This means: Your answers will be securely stored, used only for evaluation, and not shared with third parties.

If you have any questions, you can contact us at any time

**Thank you for your participation!**

**Privacy Policy**

**Privacy Statement for Survey Participation in the Scientific Study:**

**Evaluation Project "Comprehensive Evaluation of Teleconsultations in Inpatient and Outpatient Long-term Care Facilities"**

**Information on Data Handling According to Article 13 EU General Data Protection Regulation (GDPR):**

**Survey Implementation:**

The surveys are conducted online using SoSci software. Completing the survey takes approximately 10-15 minutes. We conduct the survey in a pseudonymized manner: The surveys are marked with a personal code word (pseudonym). Only you can associate this pseudonym with your person. A guide for creating the code word is presented at the beginning of the survey. It is not possible for outsiders to trace the surveys back to your person. Only project staff from the comprehensive evaluation have access to the survey data.

Participation in the survey is fundamentally voluntary. You can withdraw your consent to participate at any time without giving reasons. Withdrawal has no disadvantages for you. All your information will be treated confidentially.

The processing, use, and archiving of pseudonymized data is done on survey forms and electronic data carriers. The pseudonymized data will normally be archived for 10 years after publication, unless the purpose of the study, e.g., for inclusion in a database and for long-term studies, requires a longer storage period.

The research results from the study will be published in anonymized form in professional journals or scientific databases. Your identity will not be revealed when publishing the research results.

You can request information about your stored data at any time and request a free copy, and you have the right to have incorrect data corrected. For this, you must personally contact the researchers and provide your pseudonym, because without the pseudonym it is not clear to the processing staff who you are. You can also request at any time that your data be deleted or anonymized so that a connection to your person can no longer be established. These rights are limited under § 13 of the State Data Protection Act insofar as these rights would likely make the realization of the respective research purposes impossible or seriously impair them, and the limitation is necessary for the fulfillment of the respective research purposes. The right to information also does not exist if the data is necessary for scientific research purposes and providing information would require disproportionate effort.

The data controller according to Art. 4 para. 7 GDPR is the University Hospital Tübingen, a legal entity under public law of the University of Tübingen, Geissweg 3, 72076 Tübingen, Phone: 07071 29-0, service@med.uni-tuebingen.de. The person responsible for data processing in this study is the study director Prof. Dr. Joos. If you have questions about the use or processing of your data, please contact Sofie Wössner (sofie.woessner@med.uni-tuebingen.de).

If you have concerns or complaints regarding data protection or want to exercise your rights according to Art. 15ff GDPR, you can contact: University Hospital Tübingen, Data Protection Officer, Geissweg 3, 72076 Tübingen, Phone: 07071 29-87667, Email: Datenschutz@med.uni-tuebingen.de. You also have the right to complain to the competent supervisory authority for data protection (State Commissioner for Data Protection and Freedom of Information in Baden-Württemberg, P.O. Box 10 29 32, 70025 Stuttgart, Phone: 0711 / 61 55 41 - 716, Email: Poststelle@lfdi.bwl.de).

The legal basis for processing is Art. 6 para. 1 lit. a General Data Protection Regulation (GDPR). Your express consent by signing the data protection consent form is required for the collection, storage, use, and disclosure of your data.

**Consent Statement for Handling Data Collected in the Study**

I agree that:

- Data about me will be collected, pseudonymized, archived, and possibly pseudonymized shared on electronic data carriers as part of this study
- The pseudonymized data may be used for publication purposes (including qualification papers such as dissertations). I was assured that all personal data that would allow conclusions about my person and my employer will be deleted or anonymized
- The pseudonymized data may also be used for teaching purposes and workshops in the Institute of General Practice and Interprofessional Care or in the Institute of Health Sciences, Department of Nursing Science, limited to small excerpts. Here too, I was assured that all personal data that would allow conclusions about my person and my employer will be deleted or anonymized
- The pseudonymized data may be used for further research projects of the Institute of General Medicine and Interprofessional Care or the Institute of Health Sciences, Department of Nursing Science

I have been informed that:

- I can request information about my stored data and correction of incorrect data at any time. For this, I must provide my pseudonym because otherwise a connection to my person is not possible
- I can request at any time, for example when withdrawing from study participation, that my data collected up to that point be deleted or immediately anonymized (unless there are restrictions according to § 13 of the State Data Protection Adaptation Act or § 27 of the Federal Data Protection Adaptation Act, see above)

I declare that I have been adequately informed about the collection and processing of my data collected in this study and my rights, and I agree to the use of the data collected in this study in the form specified above.

I am aware that implementation of my data subject rights is only possible by providing the pseudonym. I have been informed that I must keep the pseudonym in a safe place.

**Contact Information:**
[…]

**Consent Options:**

☐ No, I do not want to participate in this study.

☐ Yes, I agree to the use of data collected in this study in the described form. I know that I can request at any time, for example when withdrawing from study participation, that my data collected up to that point be deleted or immediately anonymized.

**Additional Contact Information:**

**Data Protection Officer of University Hospital Tübingen**
Calwerstraße 7/4, 72076 Tübingen
Phone: 07071 29-87667
Email: dsb@med.uni-tuebingen.de

**State Commissioner for Data Protection and Freedom of Information in Baden-Württemberg**
Postal Address: P.O. Box 10 29 32, 70025 Stuttgart
Phone: 0711/615541-0, FAX: 0711/615541-15
Email: poststelle@lfdi.bwl.de

**Pseudonym Creation**

**Why is your pseudonym important?**

Your pseudonym serves to ensure your anonymity in this survey. It will be assigned exclusively to you and will not be stored anywhere else or linked to personal data.

This ensures that your answers cannot be traced back to your identity.

Please keep your pseudonym carefully, as we will repeatedly ask you to provide the pseudonym during your survey.

You can find your pseudonym and its composition in the document you received with the privacy statement for the survey.

Please keep your pseudonym carefully. You will also need it for the next survey periods.

**Create Your Pseudonym:**

**The last two letters of your mother's last name:**[____]

**The number of letters in your mother's (first) first name (e.g., 08):**[____]

**The last two letters of your father's (first) first name:**[____]

**Your own birth date (only the day, e.g., 05):**[____]

**This is your pseudonym:**[________________]

Please keep your pseudonym carefully. You will need it on the next page and for the next survey periods.

**Pseudonym**

**1. Please enter your pseudonym here:**[________________]

**Baseline Survey**

In the next questions, we're asking about the time **before teleconsultations existed.** Please tell us how you experienced that time.

**2. Before teleconsultations existed: How long did a normal on-site visit last (total time of a visit)?**

*Please give the time in minutes. Also count the time you spent preparing for and following up on the visit, for example reading or printing documents. Also count the time afterward when you wrote something down or passed information along.*

Minutes: **___________**

**How many people were cared for within this teleconsultation?**

**Number:** ___________

**3. Who was normally present during an in-person visit? Please think of all people who usually participated.**

*You can select multiple answers. If you're not entirely sure, please choose the answer that fits best.*

☐ Residents/Clients/Patients
☐ Family members and relatives
☐ Nursing professionals
☐ Physicians
☐ Medical assistants
☐ Special education and care professionals
☐ Occupational therapists
☐ Physical therapists
☐ Others

**4. Please indicate how much time was previously required to travel to the nursing home (one way/one direction, in minutes).**

**_____ minutes**

**Sociodemographic Data**

**Information About You (Sociodemographic Data)**

A few questions about you:

This is about basic information about you. Please answer the questions as best you can. It's okay if you don't know everything exactly.

**5. Name of the facility where you work (e.g., Sunshine Nursing Home, Rainbow Home Care Service, or Sunflower Medical Practice)**

[_________________________________]

**6. Please indicate your gender.**

☐ Female
☐ Male
☐ Neither category applies / No answer

**7. Please indicate your age.**

☐ 18-19
☐ 20-29
☐ 30-39
☐ 40-49
☐ 50-59
☐ 60-69
☐ 70+
☐ No answer

**8. What is your employment status?**

☐ Full-time
☐ Part-time
☐ Marginal employment ($520 basis)
☐ Other _____________________
☐ No answer

**9. What position do you hold in your work?**

☐ Practice owner/Manager
☐ Physician
☐ VERAH/NäPA (Medical Assistant with additional qualification)
☐ Medical Assistant
☐ Nursing professional
☐ Other
☐ No answer

**10. What professional training/qualifications do you have?**
*Multiple answers possible.*

☐ Physician in training
☐ Specialist physician with primary care mandate
☐ Completed degree (Bachelor, Master)
☐ VERAH/NäPA (Medical Assistant with additional qualification)
☐ Nursing professional
☐ Other

**11. How long have you been working in your profession after completing your training or studies?**

☐ Less than 3 years
☐ 3-5 years
☐ 6-10 years
☐ More than 10 years
☐ No answer

**12. Have you conducted or participated in one or more teleconsultation in the last four weeks?**

*This means: Have you conducted a teleconsultation yourself or helped with it, for example by sharing information or participating in the conversation?*

☐ Yes
☐ No
☐ No answer

**Teleconsultation Usage Data**

**Please answer the questions as best you can.**

**Your assessment should refer to the past four weeks. It's okay if the information is not completely accurate.**

**13. How many teleconsultations have you planned in the last four weeks?**

*That is: How often have you scheduled and organized an appointment for a teleconsultation? Even if you didn't conduct it yourself.*

☐ 1 ☐ 2 ☐ 3 ☐ 4 ☐ More than 5 ☐ None

**14. How often have you participated in a teleconsultation in the last four weeks?**

*That is: How often have you been present during a teleconsultation? Even if you didn't conduct it yourself.*

☐ 1 ☐ 2 ☐ 3 ☐ 4 ☐ More than 5 ☐ None

**15. What was the most common reason for a teleconsultation in the last four weeks?**

You can select multiple answers. If you're not sure, choose the answer that fits best.

☐ General, unspecific
☐ Blood and blood-forming systems
☐ Digestion
☐ Eye
☐ Ear
☐ Cardiovascular
☐ Musculoskeletal
☐ Neurological
☐ Psychological
☐ Respiratory
☐ Skin
☐ Endocrine system, metabolism, nutrition
☐ Urological
☐ Gynecological (Female genital and reproductive organs)
☐ Male genital
☐ Social problems
☐ Care process
☐ Palliative care
☐ Other

**16. Who was normally present during a teleconsultation? Please think of all people who usually participated.**

*You can select multiple answers. If you're not entirely sure, please choose the answer that fits best.*

☐ Residents/Clients/Patients
☐ Family members and relatives
☐ Nursing professionals
☐ Physicians
☐ Medical assistants
☐ Special education and care professionals
☐ Occupational therapists
☐ Physical therapists
☐ Others

**17. Please rate the following statements:**

*You can say whether you agree with the statement or not - and how strongly.*

**Leadership in your facility/practice/home care service has supported the use of the application.**

*This means: Did the bosses or managers help me use the new system? For example, through additional work time, training, or technical support. Do you feel well supported by leadership in using teleconsultations?*

| No, not at all | Rather no | I'm unsure | Rather yes | Yes, definitely |
| --- | --- | --- | --- | --- |
| ☐ | ☐ | ☐ | ☐ | ☐ |

**For the care of the person under care, the result of the teleconsultation was usually good.**

*This means, for example: The person under care received the help they needed.*

| No, not at all | Rather no | I'm unsure | Rather yes | Yes, definitely |
| --- | --- | --- | --- | --- |
| ☐ | ☐ | ☐ | ☐ | ☐ |

**18. Please rate the following statements:**

*You can say whether you agree with the statement or not - and how strongly.*

**With teleconsultations, I save time compared to standard visits.**

*This means the teleconsultation often takes less time than a normal visit with the doctor.*

| No, not at all | Rather no | I'm unsure | Rather yes | Yes, definitely |
| --- | --- | --- | --- | --- |
| ☐ | ☐ | ☐ | ☐ | ☐ |

**With teleconsultations, I lose time compared to standard visits.**

*This means the teleconsultation often takes longer than a normal visit with the doctor.*

| No, not at all | Rather no | I'm unsure | Rather yes | Yes, definitely |
| --- | --- | --- | --- | --- |
| ☐ | ☐ | ☐ | ☐ | ☐ |

At this point in the questionnaire, the UTAUT2 is used in its German translation. For the original English version, please refer to: Schomakers, E.-M., Lidynia, C., Vervier, L. S., Valdez, A. C., & Ziefle, M. (2022). *Applying an extended UTAUT2 model to explain user acceptance of lifestyle and therapy mobile health apps: Survey study*. *JMIR mHealth and uHealth, 10*(1), e27095. <https://doi.org/10.2196/27095>.

**Thank you for your participation!**

Please keep your pseudonym carefully.

You will also need it for the next survey periods.

If you have any questions or comments, please feel free to contact: […]
