# Supplementary material for: Participatory development of an evaluation and data model for teleconsultations in long-term care: study protocol based on the MRC framework
Source: BMJ Open. 2026 Jan 23;16(1):e107644. doi: 10.1136/bmjopen-2025-107644 (PMC12853425; doi:10.1136/bmjopen-2025-107644)
Supplement: online supplemental file 5 [file bmjopen-16-1-s005.docx]

# Guideline (focus group) interviews

**Time frame:** approx **30 min** online (zoom?)

**Objective** (according to evaluation concept): Experiences of interprofessional collaboration through the televisit (TV)

**Theoretical framework:** FITT model (Ammenwerth et al. 2006)

| **Topics / Categories** | **Narrative impulses / guiding questions** | **Specific requests** |
| --- | --- | --- |
| **Start (5 min)** | | |
| **Welcome and personal introduction**  **Information on the topic, procedure, organization**  **Introduction** |  | **Participants** |
| **Short round of introductions**  **(recording as minutes)**  **Participants** | - Name - Professional background - How many years in the profession - Activity in institution - Start recording after requesting consent |  |
| **Main (20 min)** |  |  |
| **Daily work routine** | **Question 1:**  Describe how you work with the TV today? | What challenges did you face at the beginning?  How did you learn to deal with the TV?  How did you manage to involve residents or customers and their relatives? |
| **Relevance/ task aspect** | **Question 2:**  Introduction: When you carry out a TV together, different professional groups come together... for example doctors and nurses.  How would you describe the cooperation with other professional groups during the TV? | How do agreements work between you and other professional groups at TV? |
| **Placeholder (feedback from stakeholders)** | **Question 3:**  Introduction: As a care professional, you can assess the benefits that your residents or clients gain from the TV.  In your opinion, what are the advantages and disadvantages of a televisit for the residents? | To what extent has the TV had an impact on transportation and admissions? |
| **End (5 min)** | | |
| **Short final round (all)** | **Question 4:**  Do you have any other fundamental suggestions? |  |
| **Thanks for your cooperation and farewell** |  |  |
