# Supplementary material for: Participatory development of an evaluation and data model for teleconsultations in long-term care: study protocol based on the MRC framework
Source: BMJ Open. 2026 Jan 23;16(1):e107644. doi: 10.1136/bmjopen-2025-107644 (PMC12853425; doi:10.1136/bmjopen-2025-107644)
Supplement: Supplementary file 6 [file bmjopen-2025-107644.reviewer_comments.pdf]

PEER REVIEW HISTORY

BMJ Open publishes all reviews undertaken for accepted manuscripts. Reviewers are asked to complete a checklist review form and are provided with free text boxes to elaborate on their assessment. These free text comments are reproduced below.

ARTICLE DETAILS

Title (Provisional)

Participatory development of an evaluation and data model for teleconsultations in long-term care: Study protocol based on the MRC Framework

Authors

Woessner, Sofie; Hahn, Laura; Kaltenbach, Christina; Joos, Stefanie; Mahler, Cornelia; Henschke, Cornelia; Koch, Roland

---

VERSION 1 - REVIEW

**Reviewer**	**1**
**Name**	**Maduekwe, Nnamdi Ifeanyi**
**Affiliation**	**National Population Commission of Nigeria, Department of Population Studies**
**Date**	**30-Oct-2025**
**COI**	**None**

The protocol is quite detailed in the coverage of subject area. Thus, though tele-consultation is an emerging and evolving field, the protocol presents a lot information on the subject.

A major challenge is inadequacy in the use language which has amongst others affected the presentation of the objectives.

Please see, for example:

Page 6, Line 12: Sentence is not complete: ‘’International Comparative….”

Page 7: Objectives not well stated and seems nebulous. For example the second objective could have stated as: To identify organizational, technical, and contextual factors influencing the implementation of teleconsultations in long-term care

In some parts of the protocol, there seems to be a problem with the tense. Since the report is for a prospective study the language should reflect the future tense for example, ‘’will be’’ instead of ‘’has been’’. or ''is used''. See for example page 8, Line 34

**Reviewer**	**2**
**Name**	**Martinussen, Mie Hjort**
**Affiliation**	**Nordsjællands Hospital, Department of Clinical Research**
**Date**	**05-Nov-2025**
**COI**	**None**

I am pleased to say that this is a well-written protocol for an interesting study. Here are my comments:

- Although you clearly state the choice to forego the term stakeholder in favor of interest-holder, the term stakeholder still appears multiple times in the manuscript (e.g., under "Study aim"). Additionally, interest-holder is defined twice (p. 6 and 9). Revising this would help reduce potential confusion.

- The figures lack titles and descriptions.

- I found Figure 1 difficult to interpret. The text is very small, and there is a lot of information in a single figure. Could it be presented differently? Furthermore, the explanation of the TM platform and messenger services should be clear from the figure alone.

- The manuscript repeatedly mentions a "participatory development process," but it is not entirely clear who participated, when they participated, and what their contributions were. Clarifying this would strengthen the manuscript.

- In the article summary, you state that the study does not aim to evaluate effectiveness. It might be beneficial to revisit this point and elaborate on it in the discussion.

VERSION 1 - AUTHOR RESPONSE

**Reviewer: 1 Dr. Nnamdi Ifeanyi Maduekwe, National Population Commission of Nigeria, Obafemi Awolowo University**<br>The protocol is quite detailed in the coverage of subject area. Thus, though tele-consultation is an emerging and evolving field, the protocol presents a lot information on the subject.	
**Reviewer comment**	**Authors' comments / revisions**
A major challenge is inadequacy in the use language which has amongst others affected the presentation of the objectives.<br><br>**Page 6, Line 12: Sentence is not complete: ''International Comparative…."**	Thank you for highlighting this issue. We revised the language throughout the manuscript to improve clarity and correctness, and we addressed the specific incomplete sentence you noted by correcting it and ensuring it is grammatically complete.<br><br>**Addition to text**:<br>*"International comparative data suggest that, to date, the German healthcare system has been only partially able to respond adequately to the growing demand for nursing care."*
Page 7: Objectives not well stated and seems nebulous.<br>For example the second objective could have stated as: To identify organizational, technical, and contextual factors influencing the implementation of teleconsultations in long-term care	Thank you very much for this helpful feedback. We adopted the suggested wording for the second study objective. In addition, we hope that the revisions made throughout the protocol help clarify how the study objectives should be understood overall**.**<br><br>**Addition to text**:<br><br>**Study Objectives**<br><br>• *To identify organizational, technical, and contextual factors influencing the implementation of teleconsultations in long term care.*
In some parts of the protocol, there seems to be a problem with the tense. Since the report is for a prospective study the language should reflect the future tense for example, ''will be'' instead of ''has been''. or ''is used''. See for example page 8, Line 34	Thank you for pointing this out. We have carefully gone through the protocol and paid attention to the tenses applied in the manuscript and have undertaken corrections.

**Reviewer 2: Ms. Mie Hjort Martinussen, Nordsjællands Hospital**<br>Comments to the Author: I am pleased to say that this is a well-written protocol for an interesting study. Here are my comments	
Although you clearly state the choice to forego the term stakeholder in favor of interest-holder, the term stakeholder still appears multiple times in the manuscript (e.g., under "Study aim"). Additionally, interest-holder is defined twice (p.	Thank you for this helpful comment on terminology. We appreciate this careful observation about terminology. We revised the text for consistency by replacing remaining instances of "stakeholder" with "interest holder" and removing the duplicate definition to reduce confusion.

6 and 9). Revising this would help reduce potential confusion.<br><br>"The participating projects are hereinafter referred to as interest-holder (22)."	**Addition to text**:<br>*"Representatives of the participating projects are referred to as interest holders (22), as defined above."*
The figures lack titles and descriptions.	Thank you for drawing attention to the figures. We added clear titles and expanded the figure legends so that each figure can be understood on its own.
I found Figure 1 difficult to interpret. The text is very small, and there is a lot of information in a single figure. Could it be presented differently? Furthermore, the explanation of the TM platform and messenger services should be clear from the figure alone.<br><br>**Figure 1. Overview of project structures.**	Thank you very much for your careful reading and for highlighting the challenges with Figure 1. We agree that the figure is complex, as it reflects the substantial heterogeneity across the seven projects and the different technical solutions in use.<br><br>To improve clarity, we revised the manuscript text on page 8, lines 3 to 12 to better explain the purpose of the figure and how it should be read. We also added a clear figure title and an expanded legend beneath Figure 1 describing the color coding, symbols, and line types. We hope these revisions make Figure 1 easier to interpret.<br><br>**Addition to Figure 1:**<br><br>**Figure 1. Overview of project structures.** Color coding indicates the care setting: blue, partially inpatient or inpatient; light yellow, outpatient; care, mixed setting; pink, health insurance provider. Light blue rectangles represent project leads. Dark blue ovals indicate key contacts relevant to implementation and data collection. Solid lines indicate direct organizational affiliation. Dashed lines indicate loose connections or connections limited to the project context. Own illustration.<br><br>**Addition to the text:**<br>*"Given the diverse technical, organizational, and institutional starting conditions across these projects, a structured visual representation was developed to support the interpretation of project contexts (see Figure 1). The figure serves as an analytical aid by illustrating how each project is embedded within*

	existing care structures and by providing an overview of key interest holders and potential data flows. It also reflects the heterogeneity of the telemedical solutions used, ranging from fully equipped systems for synchronous teleconsultation to modular platform solutions and asynchronous messenger-based formats."
The manuscript repeatedly mentions a "participatory development process," but it is not entirely clear who participated, when they participated, and what their contributions were. Clarifying this would strengthen the manuscript.	Thank you very much for this helpful suggestion. We have clarified who participated in the participatory development process by specifying the involved groups and roles, and we added this information to the Methods section (page 8, line 42 onward).<br><br>**Addition to text 3.2 Study design:**<br><br>*"The participatory interest holder analysis involved the project leads from all seven projects. Project leads also invited selected staff members from their respective project contexts, including care facility managers, project managers, registered nurses, general practitioners, administrative staff, and medical assistants, to contribute to the workshops."*
In the article summary, you state that the study does not aim to evaluate effectiveness. It might be beneficial to revisit this point and elaborate on it in the discussion:<br><br>• ***This study does not aim to evaluate effectiveness, it will systematically prepare for a future outcome-focused trial by developing core data structures and measurement tools***	Thank you for this helpful suggestion. We revisited this point and expanded the Discussion to clarify why the study does not aim to evaluate effectiveness, and how it instead focuses on the participatory development of the data model and measurement tools needed for the planned next phase of the study.<br><br>**Addition to text in Summary**:<br>*"This protocol does not aim to evaluate effectiveness. It describes the participatory development of the data model and measurement tools needed to enable a future outcome focused study."*<br><br>**Addition to Discussion text***:*<br>*"In line with this focus, the protocol does not aim to evaluate effectiveness. Rather, it details the participatory development of the data model and data collection instruments that will underpin the planned analyses in the next phase of the study."*
